# Clinical Instruction

**Published:** 1889-11

**Authors:** J. Taft


### Clinical Instruction.

BY J. TAFT.

Read before the Seventh Ohio District Dental Society, September 17, 1889.

There is a tendency in all technical departments of human occupation to higher attainments, and in the course of study and preparation more is required than formerly. This does not involve letting down and lessening the requirements in the study and attainment of principles. Indeed, the latter is now as highly appreciated and valued as ever before. The importance of technical attainments was never so highly valued as at the present time, and in this respect there is a steady growth. A constant and persistent effort is being put forth for greater things in this direction. It is a laudable effort, to solve one of the social problems of the day. The trend of the times is clearly shown by the establishment of institutions of technology, and, still further, by the introduction and establishment of systems of technical teaching as a part of the common school system, and in connection with other educational institutions as well. This change is being effected by a growing realization that the hand as well as the brain should be educated. The world needs more thinking workers, and less workers with untrained minds. This occurs from the demand for a higher skill in all the lines of human work, together with the fact that machinery has vastly reduced the demand for unskilled labor. A recent writer says, "There is room for improvement in the reduction of knowledge to practice. Children should not only be taught the use of the mind, but the use of the hand as well. There is no longer the large opportunity of former times for earning a livelihood in the ranks of unskilled labor. The inventions and improvements in the realms of machinery are reducing the demand for unskilled labor to the minimum. A knowledge of mechanics, of tools, skill in the management of machinery, knowledge of some trade, are becoming more and more necessary, in order to secure prosperity in life. The movement in this direction is in the interest of society, humanity, morality, and religion. It has been demonstrated that a great majority of criminals come from the classes untrained in labor. Idle people—often idle from necessity—are especially susceptible to temptation."

Attention is here called to the subject of technical teaching; not to criticise the present methods, but to consider whether some improvement may not be made. It will probably be admitted by all who have experience in the matter, that teaching in the technical department of our colleges is attended with far greater difficulty than in any other branch, and, especially, is this true in the Prosthetic Department. It seems here quite difficult to attain satisfactory results. Very few of those who go out from our colleges, notwithstanding they have the indorsement of eminent teachers, possess the skill sufficient to meet in a creditable manner the ever varying cases presented in an ordinary practice. Perhaps, the majority of such persons have the ability to construct an artificial denture without reference to the case to which it is to be applied; but to the principles involved in the application and adjustment, the knowledge and skill are of the minus quantity. Of the proper arrangement for comfort, utility in mastication, voice-function, restoration of features and expression, usually little or nothing is known. To this, the reply is sometimes made that all this will come by and by, after years of experience in practice. What would be thought of the surgeon who would present such an apology? Would any one of you willingly place himself in such hands? What shall be said of the average ability of the graduates of the dental colleges as to their ability as operators? Is it not true, that too often the instructors, as well as the students, are quite satisfied if there is the ability on the part of the latter to make an ordinarily good filling with the various materials that are used for the purpose? How often is it, that the student who has finished his course, performs his operations as though there were no such things or conditions as physiology, pathology, or life? But this is not because he has not given attention to, or studied these subjects, to some degree at least, but because he has failed to recognize the relation of the one to the other. This may sometimes occur because of defective teaching, but more likely because sufficient time is not devoted to the educational process. Too often, doubtless, the student arrives at the most critical and important period of his course when he is told, "Time is up," "Here is your diploma," and he is sent forth upon the public to do good or evil as circumstances may determine. Far less mischief would be done if the fresh graduate could be freed from his self-conceit and vain imagination. In many instances this comes by and by, but not until after many mislakes and blunders have been made that are cruel to the patient and a curse to the operator.

In casting about to ascertain why these things are so, the earnest inquirer will naturally turn his attention to the modes pursued in our system of education, and, first as to time.

When one looks over the curriculum of our dental colleges and makes a note of the branches embraced in them, and then endeavors to comprehend the nature of each branch, and what should be possessed in the way of preliminary preparation for entering upon the study of, and the time necessary for the mastery of these branches, is it any wonder that the exclamation, "too short, too short," forces itself upon the mind?

No one, we think, will claim, or attempt to assume, that the clinical teaching in our colleges, as a rule, is what it ought to be. For securing the best results two or three changes from the present mode of procedure is desirable. In clinical teaching the instructors should be men of extended experience, and thorough in their practical ability. The practice of trusting to mere tyros the all-important work of this instruction, is a fatal mistake. It is often the case that the clinic room is in charge, in the main, so far as instruction is concerned, of an under-graduate, with often one or two assistants, usually inferior to himself. Such instructors can not command the respect that would be given to a more experienced and skillful teacher—nor can they be expected to give so thorough instruction. The practice of securing the service of a number, more or less of what is usually denominated clinical instructors from the profession—men having no connection with the colleges, and never more than a common interest in it, except the eclat that may attach to them from such an appointment, is one the advantage of which is very questionable indeed. It has been suggested that men are sometimes selected for this purpose rather to secure their special influence than for any real benefit it may be to the student. How this may be, I will not here affirm, but upon general principles we think it may be fairly concluded that a man who has not been at all engaged in teaching, though a good practitioner he may be, can not be equal to him, or to those who make teaching their business.

It is then a question whether it is justice to the student to put him in the charge of those who are not able to give him the best instruction. Again, the best interests of the student is conserved by keeping him under a steady and uniform course of instruction until he has well mastered that which he has in hand. Placing the student and beginner in charge of a number of different individuals with little or no experience in teaching, and each, perhaps, having a method or methods differing more or less from all the others, necessarily tends to the confusion of the student in the work in hand. What would be thought of the lecturer or didactic teacher of any given branch, having five to twenty men come before his class and work in his special field? And yet this would not be as fatal to the interest of the student as the course referred to above, and too often adopted. In clinical teaching the best results can only be attained by frequent personal contact of the teacher with the pupil. He should, especially in the early part of the instruction, frequently see every operation that the student performs, and be sure that every step of every operation is thoroughly and well accomplished as the work progresses. In order to do this, only a limited number of students should be in charge of one person--fifteen to twenty-five being the limit.

---

Another Use for Benzol.—This liquid is a great solvent of oils and grease. It will clean off the old grease that has been used to lubricate a microscope joint, and leave the surface bright and clean for a fresh application of the lubricant. Benzol is very convenient for cleaning the spindle to a turn-table when the table does not run smoothly.

